# Composition and Function of Haemolymphatic Tissues in the European Common Shrew

**DOI:** 10.1371/journal.pone.0003413

**Published:** 2008-10-15

**Authors:** Daniel P. Bray, Malcolm Bennett, Paula Stockley, Jane L. Hurst, Anja Kipar

**Affiliations:** 1 Mammalian Behaviour and Evolution Group, Faculty of Veterinary Science, University of Liverpool, Neston, Cheshire, United Kingdom; 2 Department of Veterinary Pathology, Faculty of Veterinary Science, University of Liverpool, Liverpool, United Kingdom; The Rockefeller University, United States of America

## Abstract

**Background:**

Studies of wild animals responding to their native parasites are essential if we are to understand how the immune system functions in the natural environment. While immune defence may bring increased survival, this may come at a resource cost to other physiological traits, including reproduction. Here, we tested the hypothesis that wild common shrews (*Sorex araneus*), which produce large numbers of offspring during the one breeding season of their short life span, forgo investment in immunity and immune system maintenance, as increased longevity is unlikely to bring further opportunities for mating. In particular, we predicted that adult shrews, with shorter expected lifespans, would not respond as effectively as young animals to infection.

**Methodology/Principal Findings:**

We examined haemolymphatic tissues from wild-caught common shrews using light and transmission electron microscopy, applied in conjunction with immunohistology. We compared composition and function of these tissues in shrews of different ages, and the extent and type of inflammatory reactions observed in response to natural parasitic infections. All ages seemed able to mount systemic, specific immune responses, but adult shrews showed some signs of lymphatic tissue exhaustion: lymphatic follicles in adults (*n* = 21) were both smaller than those in sub-adults (*n = *18; *Wald* = 11.1, *p*<0.05) and exhibited greater levels of depletion (*Wald* = 13.3, *p*<0.05).

**Conclusions/Significance:**

Contrary to our expectations, shrews respond effectively to their natural parasites, and show little indication of immunosenescence as adults. The pancreas of Aselli, a unique lymphoid organ, may aid in providing efficient immune responses through the storage of large numbers of plasma cells. This may allow older animals to react effectively to previously encountered parasites, but infection by novel agents, and eventual depletion of plasma cell reserves, could both still be factors in the near-synchronous mortality of adult shrews observed shortly after breeding.

## Introduction

The immune system is the primary mechanism through which animals defend against parasites and pathogenic organisms. Immunity is believed to be a major factor in regulating host survival, as under natural conditions, even a mild parasitic infection may weaken an animal sufficiently to increase the chances of mortality through starvation or predation [1]. However, maintenance and up-regulation of the immune system requires energetic and nutritional resources [1], resulting in a trade-off between investment in immunity and other physiological processes, including growth and reproduction [2]–[4]. An appreciation of the extent to which hosts invest in immunity is therefore critical to understanding the strategies through which animals maximise fitness, and how trade-offs are mediated between current offspring production, longevity and future reproductive success [3], [4].

While there have been a number of studies of ecological immunology in birds and insects [4]–[6], there has been little effort to understand immune function of small mammals in this context outside of the laboratory. Here, we examined the unique haemolymphatic system of the European common shrew (*Sorex araneus*) to investigate the capacity of this short-lived mammal, restricted by a fast metabolism and extremely limited fat reserves, to defend against its unusually diverse parasite fauna, both as a young animal and an adult.

Common shrews have attracted considerable attention from both ecologists and parasitologists, and have a life history strategy characterized by a high investment in reproduction and a short life span [7]–[9]. Their life cycle takes 14 to 16 months to complete, with the first young born in mid-May [10]–12, and only one breeding season in the spring of the second year of life [9], [12], [13]. Both sexes can mate with multiple partners, and females are extremely promiscuous [14], [15]. Females can produce up to three litters of around seven offspring [10], [11], [16], with energy intake during lactation increasing to around three times the non-reproductive level [8]. Both males and females die shortly after breeding, such that there is little overlap between generations [12], [13], [17]–[19].

*S. araneus* has an unusually diverse parasite fauna, which includes ectoparasites [20]–[22], *Bartonella* and trypanosome infections [23]–[27], *Anaplasma phagocytophilum* [27], [28], and *Pneumocystis carinii* [29], as well as over 20 helminth species [30]. The extent to which shrews are able to mount immune responses to these parasites is unknown: commons shrews have a fast metabolic rate even for their small body size [31] but store very little energy as fat [32]. Lack of resources may therefore limit the capacity of *S. araneus* to mount immunological responses, particularly as reproductive adults, and parasitism has been suggested as one of the causes of mortality of adults after breeding [12], [13], [18]. Common shrews possess a large lymphatic organ, known as the Pancreas of Aselli, which may function in defence against parasites, though its exact role is unknown, and remains the subject of discussion [33]–[35]. To date, there have been no studies of spleen or bone marrow function in *S. araneus*.

We hypothesised that common shrews, which are not expected to survive beyond the first breeding season, would gain little benefit from investing their limited resources in immunity and immune system maintenance, at the expense of reproduction. Instead, we predicted that wild shrews would demonstrate only limited responses to parasites, and that their immune system would show signs of deterioration with age. The aim of the study was therefore to evaluate the capacity of sub-adult and adult shrews to mount immunological responses. We examined and compared the structure, composition and function of relevant haemolymphatic tissues including the pancreas of Aselli, in wild-caught common shrews of different ages pre and post maturation, and the extent and type of inflammatory reactions produced in response to naturally occurring parasitic infections. Light and electron microscopy were applied in conjunction with immunohistological characterisation of leukocyte populations. Contrary to our predictions, our results indicated that shrews are capable of mounting immune/inflammatory responses throughout their entire life span. While some degree of lymphatic exhaustion was obvious in adult animals (perhaps as a result of age-related changes, or reduced investment in immunity as a consequence of breeding effort), there was also evidence of some degree of compensation, in the form of storage of plasma cells particularly in the pancreas of Aselli, possibly as a defence against previously encountered parasites.

## Materials and Methods

### Animals and Tissue Processing

Forty-three common shrews (19 male, 24 female) were live-caught in Cheshire, England between September 2001 and June 2003. The work was performed with approval of and under a licence from English nature (licence number 20030767) held by PS. Shrews were classified into three age categories: 18 sub–adults, showing no sign of sexual development (11 female, 7 male), 3 animals undergoing sexual maturation (2 female, 1 male) and 22 sexually mature animals (11 female and 11 male) caught during or after the breeding season. Adult females all exhibited signs of mating, pregnancy and/or lactation. All animals appeared healthy when captured, and were killed humanely by overdose of inhalation anaesthetic (Fluothane, Schering-Plough Animal Health, UK). Animals were inspected for ectoparasites (data not presented) before full necropsy was performed and body mass (minus gastrointestinal tract) recorded. Bladder and oesophagus were removed and dissected in Hanks saline (40× magnification), with nematode and digenean parasites in both tissues counted and identified using keys [30]. Stomachs and guts were removed, weighed, stored in 10% formalin and later dissected in Hanks saline (40× magnification). Recovered helminths were identified as nematodes, cestodes or digeneans [30] and counted.

Tissue samples from all major organs from all animals were fixed in 4% buffered paraformaldehyde for 24–48 h prior to routine embedding in paraffin wax. Sections (5 µm) were stained with haematoxylin-eosin for histological evaluation. Sections from haemolymphatic tissues (spleen, pancreas of Aselli, bone marrow (sternum) and in selected cases mesenteric or mediastinal lymph nodes and thymus) were prepared for immunohistological examinations and the TUNEL method.

Samples from the pancreas of Aselli of one sub-adult common shrew were fixed in 2.5% glutaraldehyde and 4% paraformaldehyde in cacodylate buffer (pH 7.4) and subsequently embedded in epoxy resin. Semi-thin (1 µm) and thin sections were prepared and the latter examined using transmission electron microscopy.

### Labelling of leukocytes, proliferating and apoptotic cells by immunohistology and the TUNEL method

Leukocytes, proliferating and apoptotic cells in haemolymphatic tissue samples from 21 common shrews were identified using immunohistology and the TUNEL (terminal deoxynucleotidyl transferase-mediated dUTP-biotin nick end labelling of DNA fragmentation sites) method respectively. For immunohistology, monoclonal and polyclonal antibodies (cross) reacting in other species were used in conjunction with peroxidase anti-peroxidase and avidin biotin peroxidase complex methods as previously described [36]–[39]. Antibodies, their sources and detection methods are listed in Table 1.

**Table 1:** Antibodies and detection methods used to identify leukocytes and proliferating cells in the common shrew with references to suppliers and use in other species.

Cells identified	Antibody	Detection method; pre-treatment
B cells	Rat anti-mouse CD45R^a^	ABC^i^;
(predominantly undifferentiated)	(clone B220, Ly5)^b^	citrate buffer
		pre-treatment
B cells	Mouse anti-human CD79a^c^	ABC; citrate
(predominantly differentiated)	(clone HM57)^d^	buffer/EDTA
		pre-treatment
T cells	Rabbit anti-human CD3^d^^,^ e	PAP; protease pre-treatment
Monocytes/macrophages, neutrophils, myelomonocytic precursors (not 100% of cells)	Mouse anti-human	PAP; protease pre-treatment
	myeloid/histiocyte antigen^f^ (clone MAC387)^d^	
Monocytes/macrophages, neutrophils, myelomonocytic precursors («100% of cells)	Rabbit anti-human	PAP; protease pre-treatment
	Lysozyme^d^^,^ g	
Proliferating cells	Mouse anti-human	PAP; citrate buffer
	PCNA^h^ (clone PC10)^d^	pre-treatment

a [37], [51].

b Cedarlane, Hornby, Canada.

c [52].

d Dako Cytomation, Ely, Cambridgeshire, UK.

e [36], [53].

f [37].

g [53].

h [38]–[40].

i ABC- avidin biotin peroxidase complex method.

j PAP- peroxidase anti-peroxidase method.

Cellular turnover in haemolymphatic tissues was assessed by counting proliferating and apoptotic cells. Proliferating cells were identified by their expression of the proliferating cell nuclear antigen (PCNA; [38]–[40]), while apoptotic cells were demonstrated *in situ* by the TUNEL method ([39], [41]) using a commercially available kit according to the manufacturer's instructions (ApopTag™ In Situ Apoptosis Detection Kit; Chemicon, California, USA).

Consecutive tissue sections, incubated with normal rabbit or rat serum or a non-reacting mouse monoclonal antibody, were used as negative controls for polyclonal and monoclonal antibodies respectively. For TUNEL, terminal deoxynucleotidyl transferase (TdT) was replaced by distilled water on negative control slides.

All antibodies used cross-reacted with shrew leukocytes. The B cell markers CD45R and CD79a were expressed by B cells, CD45R being strongly expressed in follicular germinal centres, but relatively faintly in well-differentiated B cells within follicular mantle zones, whereas CD79a was mainly expressed by mature B cells (weak expression in germinal centres (dark zone), strong expression in the periphery of secondary follicles, positive reaction of all cells in primary follicles). Plasma cells were negative or exhibited faint staining for both B cell markers. CD3 acted as a pan T cell marker, being expressed by entire T cell zones. Both the myeloid/histiocyte antigen and lysozyme were markers for mature monocytes/macrophages and their precursors, myelomonocytic cells, as they were expressed by monocytes and macrophages and a high percentage of cells in the bone marrow. However, they seemed not to be a marker for all neutrophils, as in both the splenic red pulp and sinuses of the pancreas of Aselli, a proportion of cells with the morphology of neutrophils were negative for both antigens. PCNA-positive, proliferating cells were present in follicle germinal centres, T cell zones, bone marrow (including megakaryocytes) and splenic red pulp, the sites in the haemolymphatic tissue expected to contain proliferating cells. The TUNEL method identified cells with the morphology of apoptotic cells [39], [41] as well as apoptotic bodies (both free and within tingible body macrophages), located predominantly in follicular germinal centres.

### Assessment of lymphatic follicle and bone marrow activity, and the severity of inflammatory infiltration in the liver

Spleen and lymph node exhibited a composition very similar to laboratory mice, which allowed direct comparison for evaluation. Lymphatic follicles in the spleen and lymph nodes of all animals were classified as primary and/or secondary follicles and as small, medium or large. The presence and degree of follicle depletion (none, mild, moderate or severe) was assessed on the basis of the cellularity of the germinal centres. Large, undepleted secondary follicles were interpreted as evidence of high activity, whereas small, depleted primary follicles indicated lowest activity. Similarly, bone marrow activity was classified as low, moderate or high based on the ratio of haematopoietic cells to adipose tissue in a cross section of the marrow in the sternum.

The degree of inflammatory infiltration in the liver was assessed semi-quantitatively as mild, moderate or severe, based on the number of cells and cell layers in the portal areas or between hepatic cords.

### Statistical analysis

Statistical analyses were restricted to sub-adult and adult animals as only 3 pubescent shrews were caught. Ordinal logistic regression examined whether bone marrow activity, lymphatic follicle activity (primary/secondary follicles, presence and degree of follicular depletion) in the spleen and pancreas of Aselli and severity of inflammatory infiltration in the liver (mild, moderate or severe) varied with age class or sex. Sex and age category were entered simultaneously as independent variables into models for each dependent variable listed above, and the significance of both terms assessed using Wald tests. Mann-Whitney U tests were used to test for differences in helminth abundances between sub-adult and adult shrews.

## Results

### The spleen in adult shrews exhibits lesser activity in the white pulp but higher cellularity in the red pulp compared to the spleen in sub-adults

**Figure 1:**
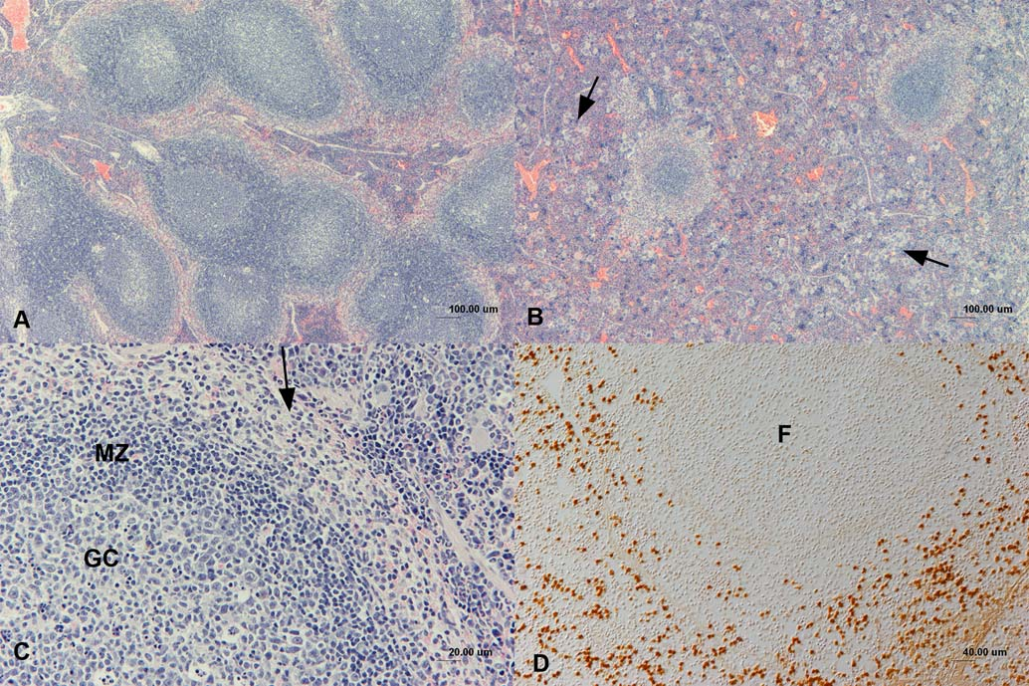
Composition of the spleen in sub-adult and adult shrews. Groups of wild-caught sub-adult and adult shrew were euthanized and the spleens examined light microscopically. Micrographs show sections of spleen. A. Sub-adult shrew. The white pulp is composed of numerous large, often interconnected secondary follicles, arranged in groups. Haematoxylin-eosin stain. B. Adult shrew. The white pulp is comprised of few, small, isolated primary follicles. The red pulp contains numerous disseminated megacaryocytes (arrow). Haematoxylin-eosin stain. C. Sub-adult shrew. Closer view of a secondary follicle. The follicle exhibits a large germinal centre (GC) and a distinct mantle zone (MZ) which is surrounded by a rim of loosely arranged macrophages and neutrophils (arrow). RP - red pulp. Haematoxylin-eosin stain. D. Sub-adult shrew. Closer view of the spleen. Very numerous cells in the red pulp and cells surrounding a follicle (F) express the myeloid/histiocyte antigen. Peroxidase anti-peroxidase method, Papanicolaou's haematoxylin counterstain.

Red and white pulp of shrews of all age groups were examined for their composition and cellular turnover. The white pulp (lymphatic follicles and T cell zones) was generally confined to the organ's centre, where follicles were arranged singly or in groups (Fig. 1). Germinal centres exhibited numerous proliferating, PCNA-positive cells (up to 50%) and few (≤5%) apoptotic cells. T cell zones were arranged around medium-sized arteries, forming periarterial lymphatic sheaths similar in size and cell density in all animals, exhibiting up to 10% proliferating, PCNA-positive cells and generally few apoptotic cells. The generally cell-rich red pulp contained neutrophils, erythrocytes and smaller numbers of lymphocytes (each <5% up to 30% T cells and mature B cells) and macrophages as well as numerous evenly distributed megakaryocytes (Fig. 1B); the red pulp exhibited some degree of cellular turnover, with approximately 10% proliferating, PCNA-positive cells and scattered apoptotic cells. Follicles and follicle groups were often delineated by a variably distinct rim of macrophages and neutrophils (Fig. 1C), together with variable numbers of mature, strongly CD79a-positive B cells, T cells and erythrocytes. In general, 30–50% of cells in the red pulp were positive for myeloid/histiocyte antigen (Fig. 1D) and lysozyme and had the morphology of monocytes/macrophages or neutrophils.

**Figure 2:**
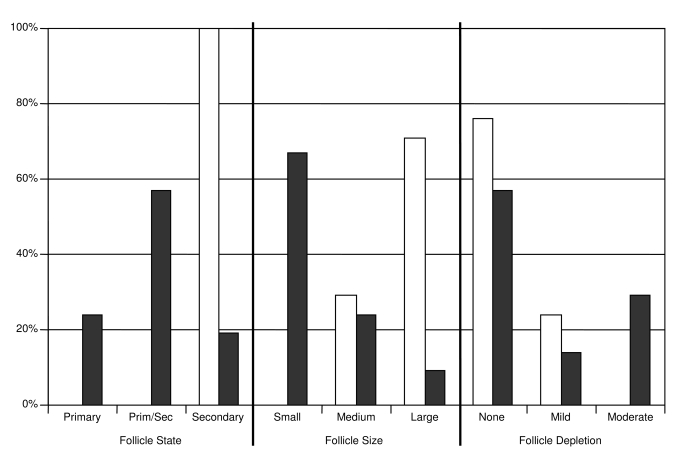
Functional state, size and degree of depletion of follicles in the spleen of common shrews. Lymphatic follicles in the spleen were classified as primary or secondary, and their size recorded as small, medium, or large. Level of follicle depletion was categorized as none, mild, or moderate on the basis of the cellularity of the germinal centres. A predominance of large, undeleted secondary follicles was taken as evidence of high activity. Bar height (y axis) shows percentage of sub-adult (open bars, *n* = 17) and adult (filled bars, *n* = 22) shrews within each category (x axis).

Age groups differed in both the composition and functional state of the spleen. Within the red pulp, the amount of neutrophils and megakaryocytes often appeared greater in adult animals than in sub-adults. The white pulp of sub-adults was exclusively comprised of secondary follicles, which often appeared interconnected and formed large groups (Fig. 1A). These follicles were for the most part large and without signs of depletion and included numerous apoptotic cells and tingible body macrophages, as well as several mitotic cells. In contrast, the majority of adults exhibited fewer follicles which were a mixture of primary and secondary follicles (Figs. 1B, 2) and generally smaller than those of sub-adults (*n* = 17, *Wald* = 17.17, *p*<0.05, Figs. 1A, 2). Follicles in adults also differed from those in sub-adults, in that they were only partially surrounded by distinct perifollicular rims. Where present, germinal centres in adults contained both mitotic as well as apoptotic cells. Follicle centres in four adults exhibited collagen deposition, and while there was a tendency for greater follicle depletion in older animals (Fig. 2), the difference between adults and sub-adults was not statistically significant (*Wald* = 2.10, not significant (ns)). The white pulp of the three pubescent animals exhibited primary and/or secondary follicles, the latter with features similar to those found in sub-adult shrews. Males (*n* = 18) and females (*n* = 21) did not differ with respect to either size (*Wald* = 0.67, ns) or depletion (*Wald* = 0.13, ns) of follicles in the spleen.

### Lymph node composition is similar in all age groups, with a relatively high proportion of plasma cells, particularly in adults

Mesenteric or mediastinal lymph nodes were examined from selected sub-adult and adult animals. All features normally associated with mammalian lymph nodes were represented: a cortex containing primary and secondary follicles, paracortex, lymphatic cords, medulla and both marginal and medullary sinuses. Compared to lymph nodes in other mammalian species [39], [42], the medulla often appeared to contain a high number of plasma cells, particularly in adults. No other differences were observed between the age groups.

### The pancreas of Aselli represents a specialised abdominal lymph node that appears to function as a plasma cell store, in particular in adult shrews

**Figure 3:**
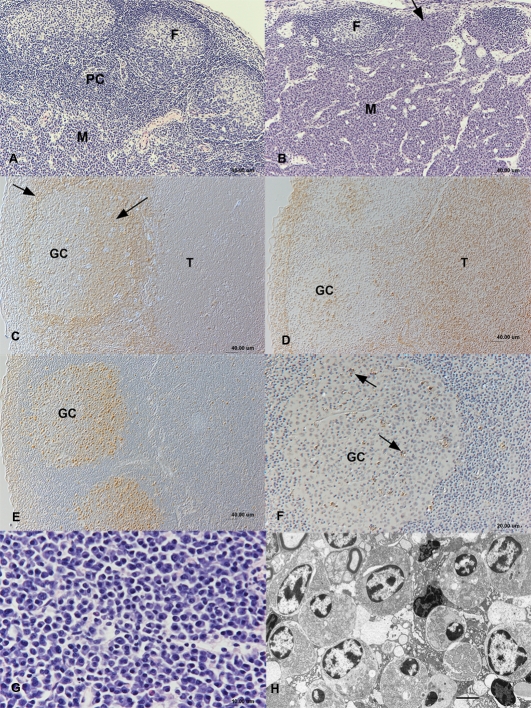
Composition of the Pancreas of Aselli in sub-adult and adult shrews. Groups of wild-caught sub-adult and adult shrew were euthanized and the Pancreas of Aselli was examined. Micrographs show sections of Pancreas of Aselli. A. Sub-adult shrew. The Pancreas of Aselli exhibits features very similar to a lymph node, with a cortex composed of large secondary follicles (F), paracortex (PC) and medulla (M). Haematoxylin-eosin stain. B. Adult shrew. The cortex is reduced to a single, secondary follicle (F) and the medulla (M) is expanded and focally extends into the cortex (arrow). Haematoxylin-eosin stain. C–F. Sub-adult shrew. Cortex and paracortex. Panel C. The B cell marker CD79a is weakly expressed in the germinal centre dark zone (GC), but expressed by B cells in the light zone and follicle mantle zone (arrows). Avidin-biotin complex method. Panel D. The T cell zones (T) are almost entirely composed of CD3-positive T cells. Within germinal centres (GC) up to 10% T cells are observed. Peroxidase anti-peroxidase method. Panel E. PCNA-positive, proliferating cells are numerous in the germinal centre (GC). Peroxidase anti-peroxidase method. Panel F. Germinal centres (GC) contain numerous apoptotic cells, often located within tingible body macrophages (arrows). TUNEL method. Papanicolaou's haematoxylin counterstain. E, F. Sub-adult shrew. Medulla. The medulla is almost entirely composed of plasma cells. Panel E: Haematoxylin-eosin stain. Panel F: Transmission electron micrograph, Bar = 5 µm.

**Figure 4:**
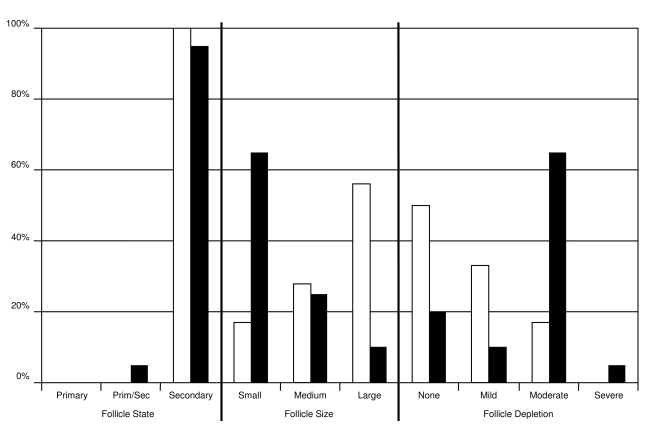
Functional state, size and degree of depletion of follicles in the pancreas of Aselli of common shrews. Lymphatic follicles in the pancreas of Aselli were classified as primary or secondary, and their size recorded as small, medium, or large. Level of follicle depletion was categorized as none, mild, moderate or severe on the basis of the cellularity of the germinal centres. A predominance of large, undeleted secondary follicles was taken as evidence of high activity. Bar height (y axis) indicates percentage of sub-adult (open bars, *n* = 18) and adult (filled bars, *n* = 21) shrews within each category (x axis).

In the past, there has been some controversy as to the composition and function of the pancreas of Aselli (lymph node-like or equivalent to the avian bursa of Fabricius [35]). The aim of this study was therefore to clarify this matter with up-to-date methodology. In general, the composition of the pancreas of Aselli was very similar to that of a lymph node. Beneath the capsule were marginal sinuses of variable width, containing disseminated lymphocytes (mostly T and B cells in equal proportions), macrophages and neutrophils, the latter either disseminated or as small accumulations. In four adult shrews, the marginal sinuses exhibited focal to extensive fibrosis. Beneath the sinuses lay a cortex containing exclusively secondary follicles (Fig. 3A–C), with the exception of one adult male where both primary and secondary follicles were present (Fig. 4). Germinal centres generally exhibited a high cellular turnover, with variable but high numbers of PCNA-positive, proliferating cells (often more than 50% of cells; Fig. 3E) and often numerous apoptotic cells (Fig. 3F). T cell zones formed a paracortex located immediately beneath the follicles (Fig. 3D) and were generally similar in size and cell density in animals of all age groups. The organ's centre (medulla) contained loosely arranged sinuses with only low numbers of macrophages. The remainder of the medulla was made up almost entirely of plasma cells (Fig. 3G, H).

In sub-adult animals the cortex generally appeared tightly packed with large follicles that exhibited no, mild or moderate depletion (Figs. 3A, 4). In adult shrews, the cortex often contained only a small number of follicles (9/20 animals; 45%), frequently with large areas of cortex devoid of follicles (5/20; 25%; Fig. 3B). Follicles occasionally seemed to extend outwards into marginal sinuses and in two animals exhibited central collagen deposition. Follicles in adults (*n* = 21) were both smaller than those in sub-adults (*n* = 18; *Wald* = 11.06, *p*<0.05, Fig. 4) and exhibited greater levels of depletion (*Wald* = 13.28, *p*<0.05, Fig. 4). No difference was found between males (*n* = 17) and females (*n* = 22) with respect to follicle size (*Wald* = 0.30, *NS*) or depletion (*Wald* = 0.42, *NS*).

Cortical areas devoid of follicles were also devoid of T cell zones. As a consequence, overall numbers of T cells in the pancreas of Aselli often seemed lower in adult than sub-adult animals. Where the cortex was devoid of follicles, plasma cells extended from the medulla to the marginal sinuses or beyond to the capsule, such that the medulla often occupied most of the organ in adult animals (Fig. 3B). Accordingly, in adult animals, the whole organ frequently appeared as an accumulation of plasma cells, surrounded by a fragmentary cortex and paracortex.

Extramedullary haematopoiesis was observed in 9/21 (43%) adult animals, as represented by scattered megakaryocytes within the outer medulla. This was not seen in sub-adult animals.

### The thymus does not show evidence of major involution in adult shrews

Thymic tissue was recovered from seven animals across all age groups. This generally consisted of a variable number of lymphocyte layers which were arranged around blood vessels and encased by a thin capsule of fibrous connective tissue. Taking the histological features of a mouse thymus in account, the thymus appeared to exhibit a variable, but generally low degree of involution in all examined shrews, regardless of age.

### The bone marrow generally exhibits moderate to high activity, regardless of age

The bone marrow generally exhibited moderate to high activity. All haematopoietic cell types known from other mammalian species were present. Approximately 10% of cells were identified as T cells, 10% as B cells (CD79a-positive, CD45R-negative; interpreted as circulating mature B cells) and 30% to 50% were myeloid/histiocyte antigen- and/or lysozyme-positive cells. At least 30% to 40% of all cells were PCNA-positive, representing relatively high levels of proliferation. No difference in bone marrow activity was found between sub-adult (*n* = 15) and adult (*n* = 16) shrews (*Wald* = 1.91, ns) or between males (*n* = 12) and females (*n* = 19; *Wald* = 0.74, ns).

### Animals of all ages can exhibit inflammatory responses to endoparasites and inflammatory changes in the liver, but no evidence of systemic non-infectious and/or neoplastic diseases

All animals harboured helminths (Table 2). The abundance of infection was greater in adults than sub-adults (Table 3). Nematodes (*Liniscus incrassatus*; Roots, 1992) recovered from the lumen and between epithelial cells of the urinary bladder were associated with mild to moderate acute to chronic lymphocyte-dominated cystitis and/or mild degeneration and sloughing of epithelial cells. Infestation of the gall bladder by the digenean *Dicrocoelium soricis* resulted in only a mild lymphocytic submucosal infiltration in one of two animals infected. Neither helminths within the gastrointestinal tract nor *Porrocaecum* sp. larvae within interscapular adipose tissue were associated with inflammatory reactions or other histological changes.

**Table 2:** Parasites identified among 43 common shrews of different ages.

Tissue	Parasites
Body cavity	Nematode larvae (*Porrocaecum* sp.^a^) and acanthocephalan larvae (*Centrorhynchus* sp.^b^)
Gall bladder	Helminths (including digenean *Dicrocoelium soricis* ^3^)
Intestine	Cestodes (*Hymenolepis* spp.^c^^,^ d and *Choanotaenia crassicolex* c ^,^ d) and nematodes (including *Parastrongyloides winchesi* c and *Longistriata spp.* c)
Kidneys	Protozoan cysts^e^
Liver	Nematodes^e^ and protozoan cysts^e^
Myocardium	Protozoan cysts^e^
Oesophagus	Digenean (*Brachylaemus fulvus* c) and nematode larvae (*Eucoleus oesophagicola* c)
Pancreas of Aselli	Nematodes (see above) and protozoan cysts^e^
Skeletal muscle	protozoan cysts^e^
Spleen	protozoan cysts^e^
Stomach	Digenea (*Brachylaemus fulvus* c), nematodes (including *Eucoleus kutori* c) and protozoan cysts^e^
Urinary bladder	Nematodes (*Liniscus incrassatus* c) and protozoan cysts^e^

a most likely *Porracaecum spirale* [30].

b most likely *Centrorhynchus* ( = *Gordiorhynchus*) *aluconis* [54], [55].

c Identified by egg morphology [30].

d [55].

e not further identified.

**Table 3:** Helminth abundances in sub-adult and adult common shrews.

	Sub-adults		Adults		*U* b
	*n* a	Median abundance	*n* a	Median abundance	
Bladder nematodes^b^	14	1.0	21	7.0	71.0*
*Porrocaecum* sp. larvae^c^	14	0.0	19	2.0	72.5*
Gut helminths^d^	13	14.0	19	50.0	42.5*

a Number of shrews examined.

b Mann-Whitney U test.

c *Liniscus incrassatus* [30].

d Encapsulated in intrascapular adipose tissue and body cavity.

e Comprising nematodes, cestodes and digeneans.

* *p*<0.05.

A high proportion of shrews (14/18 sub-adults; 78%, 2/3 pubescent animals; 67%, 12/22 adults; 55%) exhibited protozoan parasites within vessel walls that could not be further identified (Table 2). These occurred predominantly in kidneys (21/43 shrews; 49%) and myocardium (12/43 shrews; 28%), but also occasionally in the liver, the splenic red pulp, the medulla of the pancreas of Aselli and the wall of the urinary bladder. In general, these cysts did not induce any alterations apart from an occasional slight thickening of the affected vessel wall, or a mild granulomatous inflammatory infiltration. Protozoan cysts with features of *Sarcocystis* sp. were found within skeletal muscle myocytes of one adult female, without any associated reaction.

The liver of all animals exhibited a variable degree of mixed cellular (neutrophils, lymphocytes and macrophages) portal inflammatory infiltration, where T cells and B cells were present in equal amounts. No difference in the severity of the inflammatory infiltration was apparent between sub-adults (*n* = 18) and adults (*n* = 22; *Wald* = 0.03, ns), or between males (*n* = 22) and females (*n* = 18; *Wald*<0.01, ns). Infiltrates often occurred together with follicle-like accumulations of lymphocytes, which occasionally exhibited germinal centres.

Additional findings in the liver included a granuloma with central necrosis in one sub-adult, focal necrosuppurative hepatitis in two adults and variably intense (multi)focal hepatic necrosis with haemorrhage and pyogranulomatous inflammation in another three adults. In one pubescent shrew helminth parasites were observed within and outside multifocal suppurative hepatitis and haemorrhage. Five of the 43 animals exhibited granulomas within the pancreas of Aselli, with a central area of necrosis and/or mineralization (four shrews) or an embedded nematode (one animal). Focal pyogranulomatous (two adults) or suppurative inflammation (two adults), the latter in one case surrounding a nematode, were also observed. The marginal sinuses of one sub-adult shrew contained extensive focal accumulations of neutrophils, occasionally surrounding areas of necrosis.

None of the animals exhibited other major gross or histological changes. In particular there were no findings suggestive of a systemic non-infectious disease and/or a neoplastic disease.

## Discussion

While studies of laboratory animals allow aspects of immunity to be studied in controlled, repeatable environments, they may not reflect how wild animals, constrained by limited resources and at threat from a variety of infectious agents, respond to parasites and disease. We predicted that common shrews, which are short lived, store little energy as fat, and invest heavily in reproduction, would show limited responses to parasites, and their haemolymphatic tissues would deteriorate with age as expected survival decreased. Our study is the first to examine haemolymphatic tissue structure, compoosition and function in shrews using modern techniques, and one of only a small number to explore immune responses of wild animals in their natural environment.

With regards to both morphology and composition, the spleen, lymph nodes, thymus and bone marrow in *S. araneus* were found to be very similar to their equivalents in other mammalian species [39], [42]. In common with a number of species, including the musk shrew *Suncus murinus*, both bone marrow and spleen were identified as sites of haematopoiesis in *S. araneus* [43]. The results of our study also confirm that the pancreas of Aselli, which is specific to shrews, can be considered as a large, specialised lymph node [33]. The presence of a cortex with both follicles and paracortical T cell zones renders previous controversial assumptions regarding the organ's function incorrect: the pancreas of Aselli is neither a specific site of exclusive B cell production nor a functional analogue of the bursa of Fabricius in birds [35]. However, it differs from normal lymph nodes in that the centre (medulla) contains a very high proportion of plasma cells. In adulthood, the number of plasma cells and the relative size of the medulla seem to increase, until almost the entire organ is composed of plasma cells. Such a feature has not been described under physiological circumstances in any other species, and suggests that the pancreas of Aselli in *S. araneus* functions as a storage site for plasma cells, particularly in older animals. Lymph nodes in *S. araneus* were also found to contain a higher number of plasma cells than normally observed in other species [39], [42], which may emphasise a general tendency towards progressive plasma cell storage in common shrews.

In comparing the lymphatic tissues of sub-adult and adult shrews, young animals were generally found to have an activated immune system, as represented by a predominance of large, active, secondary follicles in the spleen and pancreas of Aselli. This suggests sub-adults were responding effectively to a diverse array of infectious agents, including the helminth and protozoan parasites detected in a number of tissues. Post-reproductive animals, however, exhibited characteristics indicative of immune system exhaustion: follicles were generally smaller and were often depleted, with a smaller proportion of secondary follicles, particularly in the spleen. This could indicate decreased follicular activity in adult animals, with impaired germinal centre reactions resulting in reduced B cell production [44]. Impairment of germinal centre reactions is a known feature of immunosenescence in vertebrates and has been studied extensively: in humans it has been shown to be a product of defective T cell-dependent B cell activation [44], [45]. Reduced lymphocyte production as a consequence of follicular and T cell impairment could explain why significantly lower numbers of white blood cells, and specifically lymphocytes, have been reported in old common shrews [46].

Differences between sub-adults and adults were also evident in the so-called “marginal” or “intermediate zone” of the spleen, the variably distinct rim of macrophages, lymphocytes, neutrophils and erythrocytes surrounding follicles and follicle groups seen in *S. araneus* and previously described in other species, including the musk shrew [43], [47]. This zone is considered to be the site of most intensive blood filtration in the spleen [43], [47], and its loss of integrity/intensity in older animals may indicate a reduction in filtration capacity. This might however be counterbalanced by an increase in the capacity of the peripheral phagocytic response, as represented by an increase in neutrophil numbers within both the spleen (as observed here) and the peripheral blood [46].

Both in bone marrow and splenic red pulp, the degree of haematopoiesis was similar in animals from all age groups. This concurs with similar findings in the musk shrew [43], where both the splenic red pulp and bone marrow have been identified as physiological sites of erythropoiesis, leukocytopoiesis and platelet production over the animal's lifespan. In this aspect, shrews are similar to some reptiles, whereas in other mammals the haematopoietic capacity of the spleen seems to cease after birth [43]. We also found evidence of haematopoiesis in the pancreas of Aselli in adult *S. araneus*, as demonstrated by the presence of megakaryocytes in the medulla of some individuals. Interestingly, we found no evidence of major thymic involution in *S. araneus*, even in older animals. The rate of thymic involution is known to vary between species and breeds and with intraspecific factors such as sex and diet [48], [49]. Perhaps in shrews thymic involution is delayed to maintain production of T cells into adulthood.

It has been suggested that short-lived species should limit their investment in immunity to immediate, innate responses, as the energetic costs associated with mounting specific immune reactions are unlikely to be outweighed by the benefits of increased long-term survival [1]. The dependence on innate responses may be greater for species with limited energetic reserves (such as *S. araneus*), as even a mild immune challenge is likely to result in starvation if allowed to persist for more than a short time [1]. Here, however, the presence of numerous active secondary follicles in the spleen and pancreas of Aselli, the development of small lymphatic follicles in portal areas in the liver and the generally high number of plasma cells in the pancreas of Aselli all indicate that common shrews remain consistently able to mount systemic, specific immune responses. We also observed macrophage-dominated (granulomatous) inflammatory reactions with lymphocyte involvement in both sub-adult and adult shrews, which included reactions to helminths in tissues. The increasing number of plasma cells in the medulla of the pancreas of Aselli and in lymph nodes with advancing age might even suggest a ‘refocusing’ of the immune system, from reacting to novel antigens in follicles as a young animal, to combating previously experienced parasites or pathogens with appropriate antibody responses as an adult. Plasma cells are long-lived and can survive for weeks after immunisation, particularly when not too tightly packed [50]; perhaps young common shrews invest in long term immunity by producing and storing plasma cells in the pancreas of Aselli, which can then be used to mount efficient responses against previously encountered parasites in adulthood, when reproduction places greater demands on internal resources [8]. While this strategy may allow older animals to react effectively to previously encountered parasites, infection by novel agents or eventual depletion of plasma cell reserves, could still be factors in the near-synchronous mortality of adult shrews observed shortly after the breeding season [12], [13], [18]

## References

[1] Lochmiller RL, Deerenberg C (2000). Trade-offs in evolutionary immunology: just what is the cost of immunity?. Oikos.

[2] Zuk M, Stoehr AM (2002). Immune defense and host life history.. Am Nat.

[3] Sheldon BC, Verhulst S (1996). Ecological immunology: costly parasite defences and trade-offs in evolutionary ecology.. Trends Ecol Evol.

[4] Norris K, Evans MR (2000). Ecological immunology: life history trade-offs and immune defence in birds.. Behav Ecol.

[5] Rolff J, Siva-Jothy MT (2003). Invertebrate ecological immunology..

[6] Siva-Jothy MT, Moret Y, Rolff J (2005). Insect immunity: an evolutionary ecology perspective.. Advan Insect Physiol.

[7] Stockley P, Macdonald DW (1998). Why do female common shrews produce so many offspring?. Oikos.

[8] Genoud M, Vogel P (1990). Energy-requirements during reproduction and reproductive effort in shrews (Soricidae).. J Zool.

[9] Shillito JF (1963). Field observations on growth, reproduction and activity of a woodland population of the common shrew *Sorex araneus* L.. J Zool (Lond).

[10] Middleton AD (1931). A contribution to the biology of the common shrew, *Sorex araneus* Linnaeus.. J Zool (Lond).

[11] Brambell FWR (1935). Reproduction in the common shrew (*Sorex araneus* Linnaeus).. Philos Trans R Soc Lond, B, Biol Sci.

[12] Michielsen NC (1966). Intraspecific and interspecific competition in the shrews *Sorex araneus* L. and *Sorex minutus* L.. Arch Neerl Zool.

[13] Pernetta JC (1977). Population ecology of British shrews in grassland.. Acta Theriol.

[14] Stockley P, Searle JB, Macdonald DW, Jones CS (1993). Female multiple mating-behavior in the common shrew as a strategy to reduce inbreeding.. Proc R Soc B.

[15] Stockley P, Searle JB, Macdonald DW, Jones CS (1996). Correlates of reproductive success within alternative mating tactics of the common shrew.. Behav Ecol.

[16] Crowcroft W (1957). The Life of the Shrew.

[17] Adams LE (1910). A hypothesis as to the cause of the autumnal epidemic of the common and the lesser shrew, with some notes on their habits.. Manchester Mem.

[18] Buckner CH (1969). Some aspects of the population ecology of the common shrew, *Sorex araneus*, near Oxford, England.. J Mammal.

[19] Churchfield S (1980). Population-dynamics and the seasonal fluctuations in numbers of the common shrew in Britain.. Acta Theriol.

[20] Smit FGAM (1957). Handbooks for the Identification of British Insects.

[21] Randolph SE (1975). Seasonal dynamics of a host-parasite system - *Ixodes trianguliceps* (Acarina Ixodidae) and its small mammal hosts.. J Anim Ecol.

[22] Churchfield S (1990). The Natural History of Shrews.

[23] Hoyte HM (1956). *Grahamella* (Rickettsiales) in the common shrew *Sorex araneus*.. Parasitology.

[24] Laakkonen J, Haukisalmi V, Merritt JF (1998). Blood parasites of shrews from Pennsylvania.. J Parasitol.

[25] Laakkonen J (2000). Microparasites of three species of shrew from Finnish Lapland.. Ann Zool Fennici.

[26] Holmberg M, Mills JN, McGill S, Benjamin G, Ellis BA (2003). *Bartonella* infection in sylvatic small mammals of central Sweden.. Epidemiol Infect.

[27] Bray DP, Bown KJ, Stockley P, Hurst JL, Bennett M (2007). Haemoparasites of common shrews (*Sorex araneus*) in Northwest England.. Parasitology.

[28] Liz JS, Anderes L, Sumner JW, Massung RF, Gern L (2000). PCR detection of granulocytic Ehrlichiae in *Ixodes ricinus* ticks and wild small mammals in western Switzerland.. J Clin Microbiol.

[29] Laakkonen J (1995). Characterization of *Pneumocystis carinii* infection in *Sorex araneus* - a review.. Mammalia.

[30] Roots CD (1992). Morphological and Ecological Studies on the Helminth Parasites of British Shrews [Ph.D. Thesis]: University of London..

[31] Nagel A, Merritt JF, Kirkland GL, Rose RK (1994). Metabolic rates and regulation of cardiac and respiratory function in European shrews.. Advances in the Biology of Shrews.

[32] Churchfield S (1981). Water and fat contents of British shrews and their role in the seasonal changes in body weight.. J Zool.

[33] Holmes RL (1965). Abdominal lymphoid tissue in the shrew.. J Anat.

[34] Twigg G, Hughes D (1970). The 'pancreas of Aselli' in shrews.. J Zool.

[35] Tsiperson VP (1997). Pancreas of Aselli in some species of the shrews (*Sorex araneus* and *Neomys fodiens*) as an analogue of the bursa of Fabricius in birds.. Cell Biol Int.

[36] Milner RJ, Pearson J, Nesbit JW, Close P (1996). Immunophenotypic classification of canine malignant lymphoma on formalin-mixed paraffin wax-embedded tissue by means of CD3 and CD79a cell markers.. Onderstepoort J Vet Res.

[37] Kipar A, Bellmann S, Kremendahl J, Kohler K, Reinacher M (1998). Cellular composition, coronavirus antigen expression and production of specific antibodies in lesions in feline infectious peritonitis.. Vet Immunol Immunopathol.

[38] Kipar A, Köhler K, Leukert W, Reinacher M (2001). A comparison of lymphatic tissues from cats with spontaneous feline infectious peritonitis (FIP), cats with FIP virus infection but no FIP, and cats with no infection.. J Comp Path.

[39] Köhler K, Kipar A, Reinacher M (2000). Immunohistological evalutation of haemolymphoid tissue activity in the cat.. Eur J Vet Pathol.

[40] McCormick D, Hall PA (1992). The complexities of proliferating cell nuclear antigen.. Histopathology.

[41] Gavrieli Y, Sherman Y, Ben-Sasson SA (1992). Identification of programmed cell death in situ via specific labeling of nuclear DNA fragmentation.. J Cell Biol.

[42] Wünschmann A, Kremmer E, Baumgartner W (2000). Phenotypical characterization of T and B cell areas in lymphoid tissues of dogs with spontaneous distemper.. Vet Immunol Immunopathol.

[43] Fukuta K, Nishida T, Mochizuki K (1982). Light and electron microscopic observations of the spleen in the musk shrew, *Suncus murinus*.. J Anat.

[44] Herrera E, Martinez AC, Blasco MA (2000). Impaired germinal center reaction in mice with short telomeres.. EMBO J.

[45] Fernandez-Gutierrez B, Jover JA, De Miguel S, Hernandez-Garcia C, Vidan MT (1999). Early lymphocyte activation in elderly humans: impaired T and T-dependent B cell responses.. Exp Gerontol.

[46] Wolk E (1981). Seasonal and age-changes in leukocyte indexes in shrews.. Acta Theriol.

[47] Snook T (1950). A comparative study on the vascular arrangements in mammalian spleens.. Am J Anat.

[48] Gruver AL, Hudson LL, Sempowski GD (2007). Immunosenescence of ageing.. J Pathol.

[49] Lustig A, Weeraratna AT, Wood WW, Teichberg D, Bertak D (2007). Transcriptome analysis of age-, gender- and diet-associated changes in murine thymus.. Cell Immunol.

[50] Sze DMY, Toellner KM, Garcia de Vienuesa C, Taylor DR, MacLennan ICM (2000). Intrinsic contraint on plasmablast growth and extrinsic limits of plasma cell survival.. J Exp Med.

[51] Butcher EC, Reichert RA, Coffman RL, Nottenburg C, Weissman IL (1982). Surface phenotype and migratory capability of Peyer's patch germinal center cells.. Adv Exp Med Biol.

[52] Chu PG, Arber DA (2001). CD79: a review.. Appl Immunohistochem Mol Morphol.

[53] Kipar A, Baumgärtner W, Vogl C, Gaedke K, Wellman M (1998). Immunohistochemical characterization of inflammatory cells in brains of dogs with granulomatous meningoencephalitis.. Vet Pathol.

[54] Ewald JA, Crompton DW, Johnson I, Stoddart RC (1991). The occurrence of *Centrorhynchus* (Acanthocephala) in shrews (*Sorex araneus* and *Sorex minutus*) in the United Kingdom.. J Parasitol.

[55] Roots CD, Lewis JW, Churchfield JS (1994). The morphology of hymenolepidid and dilepidid cestodes from common and pygmy shrews (Soricidae) in Southeast England.. J Helminthol.

